# Severe Avian Influenza A H5N1 Clade 2.3.4.4b Virus Infection in a Human with Continuation of SARS-CoV-2 Viral RNAs

**DOI:** 10.1155/2024/8819622

**Published:** 2024-05-27

**Authors:** Huiyan Yu, Ke Jin, Songning Ding, Ke Xu, Xian Qi, Junjun Wang, Qigang Dai, Haodi Huang, Chaoqi Xu, Shenjiao Wang, Fei Deng, Jun Li, Liguo Zhu, Changjun Bao

**Affiliations:** ^1^ Department of Acute Infectious Disease Control and Prevention Jiangsu Provincial Center for Disease Control and Prevention NanjingChina; ^2^ Department of Infectious Diseases The First Affiliated Hospital with Nanjing Medical University NanjingChina; ^3^ Nanjing Municipal Center for Disease Control and Prevention NanjingChina; ^4^ Nanjing Gulou Center for Disease Control and Prevention NanjingChina

## Abstract

**Background:**

Since 2020, global attention has heightened towards epidemics caused by avian influenza A H5N1 virus of clade 2.3.4.4b in birds and mammals. This study presents the epidemiological history, clinical manifestations, and prognosis of a unique case infected with avian influenza A H5N1 clade 2.3.4.4b, along with the continuation of SARS-CoV-2 viral RNAs, in Eastern China.

**Methods:**

We collected and analysed the patient's clinical, epidemiological, and virological data. Both sputum and bronchoalveolar lavage fluid (BALF) samples were subjected to real-time RT-PCR to test for respiratory pathogens of interests, including SARS-CoV-2 and influenza virus. Influenza virus isolation and propagation were performed on embryonated eggs. Serological tests were used to determine the presence of SARS-CoV-2 antibodies. Phylogenetic analysis was constructed to explore viral evolution and origin of A/H5N1 virus.

**Results:**

A 53-year-old female farmer with chronic bronchiectasis was hospitalized with severe pneumonia. Real-time RT-PCR revealed the presence of avian influenza A H5N1 and SARS-CoV-2 in BALF and sputum samples. Sequence analyses classified the human isolate as clade 2.3.4.4b of avian influenza A H5N1. The amino acid motif GlnSerGly at residues 226–228 of the haemagglutinin protein indicated avian-like receptor binding preference. Epidemiological investigation established that the patient had exposure to sick or dead poultry 3 days before illness onset, while no cases of human-to-human H5N1 virus transmission were identified in 31 close contacts.

**Conclusion:**

We presented that the clade 2.3.4.4b H5N1 avian influenza virus has the potential to cross-infect humans with serious symptoms, especially in individuals already affected by COVID-19. It is indeed crucial to closely monitor the virus's evolution in both avian populations and humans. Continued research and surveillance efforts are vital to monitor any potential changes in the virus, as well as to inform public health policies and interventions.

## 1. Introduction

The outbreak in birds caused by avian influenza A H5N1 virus was initially confirmed in Scotland in 1959 [1]. The first recorded outbreak of human infection with the avian influenza A H5N1 virus occurred in Hong kong in 1997, where a total of 18 cases were detected, resulting in six fatalities [2]. From January 2003 to April 2023, there have been 873 reported cases of human infection with the avian influenza A H5N1 virus globally. Among these cases, 458 were fatal, resulting in a case fatality rate of 52.5% [3]. Recent studies have shown that the high fatality rate of avian influenza virus infections is a consequence of an overactive inflammatory response and the severity of infection is closely related with virus-induced cytokine dysregulation [4].

High pathogenic avian influenza (HAPI) A H5N1 viruses (A/goose/Guangdong/1/96, Gs/GD) were first isolated from a domestic goose in Guangdong Province, China, in 1996. Since then, these viruses have undergone extensive genetic divergence. In 2008, a new clade of H5Ny viruses (including N2, N3, N5, N6, and N8), known as clade 2.3.4.4, emerged in China. From 2014 onwards, eight HA subclades of the clade 2.3.4.4 H5Ny viruses have been identified in various regions, including Asia, Europe, Africa, and North America. These subclades are denoted as 2.3.4.4a to 2.3.4.4 hr [5]. Since October 2020, an avian influenza epidemic caused by the clade 2.3.4.4b strain of the H5N1 virus has been spreading across Eurasia, Africa, and North America [6].

Although the World Health Organization (WHO) assessed the risk of the clade 2.3.4.4b of A H5N1 virus was low for human beings [7], there have been instances of infection in both wild birds and poultry during the same season. Additionally, the virus has spilled over from birds and poultry to mammal animals such as foxes [8], dolphins [9], seals [10], and others [11, 12]. As of 1 April 2023, seven human cases of infection with the clade 2.3.4.4b of A H5N1 virus have been reported worldwide. Among them, four cases reported as asymptomatic or with mild symptoms are mostly related to male workers involved in culling activities and wearing personal protective equipment (PPE). The other three cases, including this case, reported with severe or fatal outcome, have all been women exposed without personal protective equipment to sick or dead backyards poultry. None of these case reports mentioned their COVID-19 infection history [13]. Although minor mutations associated with increased virulence in mammals have been detected, no significant mammalian adaptive substitutions, insertions, or deletions have been identified [14]. Here, we reported a severe human case infected with the clade 2.3.4.4b avian influenza A H5N1 in China, described the clinical epidemiological and virological aspects, and discussed the importance of surveillance in the post-COVID-19 era.

## 2. Methods

### 2.1. Case Finding

Since 2012, an active surveillance network was established in Jiangsu Province, China, to monitor hospitalized pneumonia patients with unknown causes. In this surveillance network, patients admitted to local hospitals with a diagnosis of pneumonia or severe pulmonary infection without a clear aetiology are required to be reported to the local Centers for Disease Control and Prevention. These patients typically have a normal range or decreased white blood cell counts. As part of the surveillance process, respiratory tract-related specimens from these patients are collected and sent for pathogen analysis. The primary focus of analysis is on influenza viruses and coronaviruses, as these are known to be common causes of respiratory infections.

### 2.2. Epidemiological Investigation and Specimen Collection

A standard epidemiological investigation questionnaire was used by epidemiologists and clinicians to collect a range of important information, including demographic data, past medical history, clinical underlying diseases, history of contact with poultry or other animals (particularly those that are sick or dead), recent visits to live poultry markets, and history of influenza and COVID-19 vaccination. Information about clinical manifestations, chest imaging findings, antiviral treatment, clinical complications and outcomes, and clinical laboratory testing results were also gathered through interviews and medical record checks. To assist with diagnosis and monitoring, bronchoalveolar lavage fluid (BALF), sputum, and pharyngeal swab specimens were collected form the patient. Additionally, acute and convalescent blood specimens were also collected to help evaluate immune response and disease progression.

Close contacts were defined as individuals who have provided care, lived with, or potentially been directly exposed to respiratory secretions or bodily fluids of the patient in the 10 days before illness onset. These close contacts include the accompanied family members, other patients in the same ward, doctors, nurses who provided care to the patient without effective personal protection, and other visitors. Close contacts were required to undergo medical observation for 10 days from the date of last contact with the patient. If no signs or symptoms related to respiratory disease were present, throat swabs were collected on the 10th day to detect influenza type A viruses.

### 2.3. Molecular Test

Nucleic acids were extracted using the viral DNA and RNA Kit from Tianlong Biotechnology Co. Ltd. in Suzhou, China, and the Rneasy Mini Kit from Qiagen in Germany, following the manufacturer's instructions. These kits are commonly used for extracting nucleic acids from a variety of biological samples in molecular biology research and diagnostics. To detect influenza viruses and SARS-CoV-2, Real-time RT-PCR Kits from Bioperfectus Technology Co. Ltd. in Taizhou, China were used. These kits are designed specifically for the detection of various subtypes of influenza viruses, including seasonal influenza virus (H1, H3, and B), as well as other subtypes such as avian H5N1, H5N6, H5N8, H7N9, and H9N2, and swine H1N1 [15]. Multiple pathogen testings used liquid chip technique for common pathogens that can cause pneumonia in humans, including respiratory syncytial virus, respiratory adenovirus, seasonal influenza type A and B viruses, parainfluenza viruses, mycoplasma pneumonia, chlamydia pneumonia, and legionella.

### 2.4. Virus Isolation

Sputum and bronchoalveolar lavage fluid (BALF) samples were collected from patients and stored in a viral transport medium at 4°C. To culture the virus, 200 *μ*L from the patient sample was inoculated into the allantoic sac of 9–11-day-old specific pathogen-free (SPF) embryonated chicken eggs. The eggs were then incubated for 48–72 hr at 37°C in a biosafety level 3 (BSL-3) facility, which is a high-containment laboratory designed to safely handle infectious agents that can cause severe or potentially lethal disease.

### 2.5. Sequence Analysis

Sequencing of the isolated viral strain was conducted using the Illumina Iseq platform, following the previously described protocol [15]. The obtained sequences were then analysed by BLASTn, which compares the sequences against databases such as GenBank and the Global Initiative on Sharing All Influenza Data (GISAID). The reference nucleotide sequences of the influenza virus were downloaded from relevant databases for phylogenetic analysis. For the phylogenetic analysis, the neighbour-joining method was employed using Mega6.1 software, which is widely used for genetic analysis. This method constructs a tree based on the genetic distances between sequences and generates a phylogenetic tree representing the evolutionary relationships [16]. Sequence alignment was performed using ClustalX 1.83, a popular tool for aligning multiple sequences and identifying conserved regions or variations among them. To evaluate the reliability of the phylogenetic tree, Bootstrap analysis with 1,000 replications was conducted.

### 2.6. Serological Test

Sera samples were collected from the individuals and tested for specific antibody levels against SARS-CoV-2 using a magnetic chemiluminescence enzyme immunoassay (EIA) kit produced by Bioscience Co., Chongqing, China [17]. These antibody levels measured include IgG, IgM, IgA, and neutralizing antibodies.

## 3. Results

Based on the information provided, the patient in question was a 52-year-old female farmer with no history of smoking or drinking, who had chronic bronchiectasis and pulmonary nodules, and underwent appendicitis surgery earlier in 2022. Her BMI index was about 16.7, which falls below the normal range of 18.5–23.9. It was noted that she was inoculated neither COVID-19 vaccine nor influenza vaccine in the past year. On December 20, 2022, the patient developed a fever of 37.5°C and a cough with a positive self-test SARS-CoV-2 antigen result. The patient did not seek medical attention, and her fever lasted for 3 days, while her cough persisted for 7 days, and then she recovered without experiencing any other symptoms.

On January 31, 2023 (day 0), the patient developed fever with the highest body temperature at 39°C. Then she sought medical attention from a village doctor and was given antibiotics but did not show improvement in her condition. On February 4 (day 4), she visited a municipal hospital where she was diagnosed with a lung infection and found to have increased levels of C-reactive protein and procalcitonin. Treatment with anti-infection medication was initiated. Due to the continuous deterioration of her condition, on February 7 (day 7), she was admitted to the emergency department of a tertiary hospital with a primary diagnosis of acute hypoxia, respiratory failure, pleural effusion, hypoproteinaemia (low protein levels), and splenomegaly (enlarged spleen). Despite receiving antibiotics and non-invasive ventilation, her condition continued to worsen, and she was switched to invasive ventilation on the same night.

**Figure 1:**
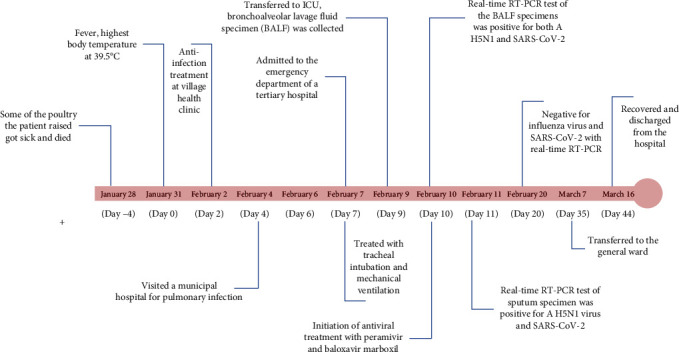
Timeline of the clinical course and key indicators of patient co-infection with A H5N1 clade 2.3.4.4b virus and SARS-CoV-2.

On February 10 (day 10), she was suspected to be co-infected with avian influenza subtype A virus and SARS-CoV-2 and transferred to an intensive care unit (ICU), where antiviral drugs for influenza (peramivir injection and baloxavir marboxil tablets) and COVID-19 (Paxlovid) were initiated with standard doses and mode of administration. Additionally, she was given norepinephrine due to going into shock.  Figure 1 showed the timeline of the clinical course and key indicators of the patient.

**Figure 2:**
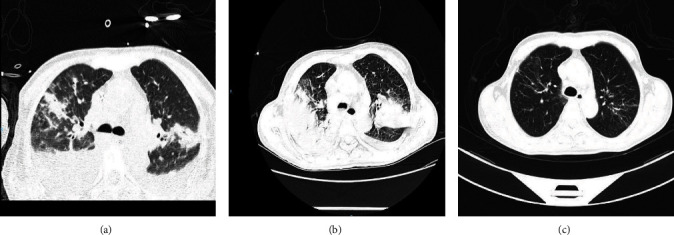
Imaging findings of a patient with co-infection of avian influenza A H5N1 clade 2.3.4.4b and SARS-Cov-2. Chest CT scans (a–c) of a patient with co-infection of avian influenza A H5N1 clade 2.3.4.4b and SARS-Cov-2 were conducted on day 7, day 15, and day 31 after illness onset, respectively. (a) Consolidation can be seen from the left picture which took on the date of admission to the emergency department of a tertiary hospital. (b) Lung lesions were slightly absorbed but with increased bilateral pleural effusions with atelectasis on day 15. (c) Chest computed tomography was re-examined 5 weeks later (on March 13), and pulmonary inflammatory lesions and pleural effusion were significantly absorbed.

On admission, the patient's white blood cell (WBC) count was within the normal range. However, there was an increased proportion of neutrophils and a decreased proportion of lymphocytes. As the patient was transferred to the ICU, the WBC count increased beyond the normal range (Table 1). No pathogenic bacteria or fungus were found in the blood culture on February 7 (day 7). The chest computed tomography (CT) performed on admission (day 7) revealed multiple patchy shadows in bilateral lungs, consolidation in the upper and right middle lobes, bilateral pleural effusions with atelectasis, and multiple enlarged lymph nodes located in the mediastinum and both armpits with a larger short diameter of about 13 micrometres (Figure 2(a)). One week after admission (day 15), the lung lesions showed slight absorption, but there was an increase in bilateral pleural effusions with atelectasis (Figure 2(b)). Five weeks later (day 41), pulmonary inflammatory lesions and pleural effusion were significantly absorbed (Figure 2(c)). The patient was transferred from the ICU to a general ward on March 7 (day 35) and then recovered fully and discharged from hospital on March 16, 2023 (day 44), after sportive and symptomatic treatment and use of antiviral drugs.

The patient did not have a recent history of travelling outside her residential city, visiting live poultry markets, or contacting with individuals with similar symptoms in the 10 days prior to her illness onset. The patient raised poultry (about 20 chickens and 10 ducks) in a semi-open poultry house in her family backyard, where wild birds would frequently visit for food. She fed and cleaned the poultry house without using any personal protective equipment. Prior to the onset of her illness, the patient administered antibiotics to the sick poultry with her bare hands. Subsequently, approximately 10 chickens died on January 28, 2023. A total of 31 close contacts were identified, including 29 doctors and nurses who provided medical care to the patient, one patient in the same ward, and the patient's husband. None of the close contacts developed respiratory symptoms during the 10-day medical observation period. On the last day of the observation period, pharyngeal swabs were collected from the close contacts and tested negative for avian influenza type A virus using real-time RT-PCR.

The BALF specimen collected from the patient on February 9 (day 9) was tested positive for both A H5N1 avian influenza and SARS-CoV-2 by means of real-time RT-PCR. The Ct values for the HA and NA genes of H5N1 were 25 and 26, respectively. The Ct values for the ORF1ab and N genes of SARS-CoV-2 were 36 and 37, respectively. The sputum specimen collected from the patient on February 11 (day 11) was still tested positive for H5N1 with a litter higher CT value (HA = 28, NA = 30). SARS-CoV-2 was barely detected in this specimen (ORF1ab = 37, *N* = negative). Both the BALF and sputum specimens tested negative for seasonal influenza viruses (H1, H3, and B) and other avian influenza virus subtypes (H5, H7, and H9). To exclude the possibility of the patient co-infection with other respiratory pathogens, multiple pathogens testing using liquid chips technique showed negative results for common pathogens that can cause pneumonia in humans, including respiratory syncytial virus, respiratory adenovirus, seasonal influenza type A and B viruses, parainfluenza viruses, mycoplasma pneumonia, chlamydia pneumonia, and legionella. The pharyngeal swab collected from the patient on February 20 was negative for both influenza virus and SARS-CoV-2 with real-time RT-PCT.

To differentiate whether the infection of the patient with SARS-CoV-2 was a reinfection or relapse, paired serum specimens were used to test the levels of several antibodies to SARA-CoV-2. The antibody titres for IgG, IgM, IgA, and neutralization antibodies against SARS-CoV-2 from the sera collected on February 20 (day 20) (7.61, 0.42, 0.39, and 9.39, respectively) did not show a significant increase compared to those results from the sera on February 10 (day 10) (4.37, 0.16, 0.16, and 9.69 respectively).

The deep sequencing data from the BLAF specimen collected from the patient on February 9 (day 10) revealed an overwhelming detection of avian influenza A H5N1. A virus was successfully isolated from the BLAF specimen, which was designated as A/Jiangsu/NJ210/2023(H5N1) (JS210). The whole genetic sequences of the virus have been uploaded to the GISAID (Access no. EPI1868382–EPI1868389). All eight genomic segments of JS210 shared the highest nucleotide acid sequence similarity with those of H5N1 isolates circulating in Eurasia, especially in Southern China since 2021 (99.1%−99.7%). The HA gene of JS210 were closely related to those of reference A/goose/Hunan/SE284/2022(H5N1) and A/chicken/Khabarovsk/24-12V/2022(H5N1) (99.7% and 99.6% similarity, respectively), and the NA gene of JS210 was closely related to those of reference A/goose/Hunan/SE284/2022(H5N1) and A/*Cygnus columbianus*/Hubei/121/2021 (H5N1) (99.2%) (Table 2).

**Figure 3:**
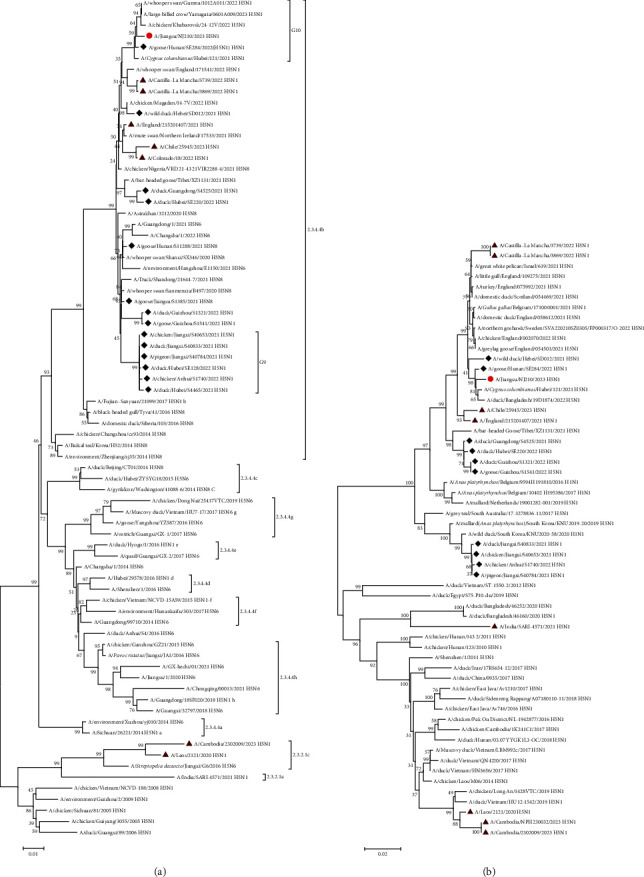
Phylogenetic tree of the full-length haemagglutinin (a) and neuraminidase genes (b) of the H5N1 bearing the clade 2.3.4.4b virus. The phylogenetic tree of the HA (a) and NA (b) coding nt sequences was generated by molecular evolutionary genetic analysis (MEGA) version 6.1 by neighbour-joining method with 1,000 bootstrap replicates. The red circle indicates human H5N1 virus isolated that we identified in 2023. The orange triangles indicate human H5N1 virus isolated in the world, 2020–2023. The black diamonds showed H5N1 viruses from environment in China, 2021–2022.

We further constructed phylogenetic trees with reference viruses of human and animal from the public datasets of Genbank and GISAID. In the HA tree, JS210 was classified into clade 2.3.4.4b, which clustered with other H5N1 strains isolated during 2021–2023 in East Asian, especially in Southern China (Figure 3(a)). Similarly, NA and six internal genes (PB2, PB1, PA, NP, M and NS) also belonged to the H5N1 isolates, which have been circulating within wild birds and poultry in East Asian since 2021 (Figure 3(b) and *Supplementary 1*). These findings indicated that JS210 might be derived from some clade 2.3.4.4b H5N1 viruses, which have been circulating in wild birds or poultry in Southern China since 2021.

The analysis of HA proteins in JS210 revealed the presence of a QSG amino acid motif at positions 226–228 (H3 numbering). This suggested that the virus has a preference for avian 2,3-linked sialic acid receptors, which are commonly found in birds, rather than mammal/human 2,6-linked sialic acid receptors [18]. Previous studies demonstrate that the mutation in HA at Q226L caused a trans/cis conformational switch in the glycan receptor that affected atomic contacts within the receptor binding site (RBS) and hence altered binding affinity [19]. The Leu at position 226 caused a shift of the 220-loop, resulting in a widening of the RBS, favourable binding to the human receptor, and abolished binding to the avian receptor. This shift and the widening of the RBS were maximal if both 226-Leu and 228-Ser were present [20]. Additionally, the HA cleavage site of JS210 contains a multiple basic amino acids motif (PPREKRRKR↓G). This motif is indicative of potential high pathogenicity to chickens [21]. Furthermore, no mutations associated with resistance to antiviral drugs, like oseltamivir, zanamivir, and adamantine, were recognized in NA and M2 proteins of JS210 (*Supplementary 2*). This indicated that the virus remains susceptible to these antiviral treatments commonly used for influenza infections. Importantly, no other significant mammalian substitutions such as E627K in PB2, D701N in PB2, and D92E in NS1 have been identified in JS210 (*Supplementary 2*). These specific amino acid changes are often associated with adaptive infection in humans. All of these mutations increase viral polymerase activity in mammalian cell lines for multiple Avian influenza virus subtypes. Combinations of these mutations, such as E627K/D701N, can also further enhance viral polymerase activity, replication, and virulence [18, 21]. Those absences suggested that JS210 may not possess enhanced capabilities for human-to-human transmission at this stage.

## 4. Discussion

We report a human case infection with the clade 2.3.4.4b avian influenza A H5N1 virus. This patient was an old female farmer with multiple comorbidities. The patient was also SARS-CoV-2 positive after having COVID-19 on December 20, 2022. Our study provides insights into the significance of surveillance for increased risk of H5N1 viruses infection among people with post-COVID-19 condition.

The Chinese people experienced an intensive epidemic of SARA-CoV-2 from the middle of December 2022 to the early of January 2023. For this patient, it is obviously that the infection with SARA-CoV-2 was prior to the infection of A H5N1 virus. Epidemiological investigation showed that the patient developed fever and cough on December 20, 2022. The self-test antigen against SARS-CoV-2 was positive. Thus, the patient was assumed to had been infected with SARS-CoV-2 at that time. When she was exposed to sick and dead poultry, she might be in the convalescent stage but in a status of a lingering virus. The positive results of IgG antibodies against SARS-CoV-2 from the sera indicate previous infection, which is consistent with the positive antigen test result [22]. Prior infection with SARS-CoV-2 might lead to the patient in a non-full competent immune state, which might play a role in increasing her susceptibility to avian influenza virus. In addition, the status of the patient including emaciated body with a BMI less than 17 and multiple comorbidities might also contribute to her infection with avian influenza virus and prolonged SARS-CoV-2 viral shedding [23].

The patient described here was infected with the clade 2.3.4.4b of avian influenza A H5N1 with full-length genes. The case was officially reported to the WHO on February 24, 2023, and the eight genes of the virus were deposited in GISAID on March 3, 2023. No human cases with A H5N1 infection were reported in mainland of China from 2015 to 2021. However, a human case with A H5N1 virus infection was notified from Guangxi, a province in Southern China in October 2022. Partial sequence of HA indicated that the virus belonged to clade 2.3.4.4b [12]. However, no live virus was isolated successfully, and no sequences were available in the database of GISAID and GenBank. As a result, it is very difficult to make a comprehensive scientific evaluation on the origin and genetic characteristics of the virus. Clade 2.3.4.4b of A H5N1 was first identified in 2005 in poultry farms in the Netherlands and then spread to other countries in the continents of Eurasia and America, becoming the primary virus causing outbreaks in poultry and wild birds worldwide nowadays [6, 10]. The clade 2.3.4.4b of avian influenza virus H5N1 started to be detected in wild birds and poultry in the south of China in 2021 [24]. Before this avian influenza virus, H5N6 virus bearing clade 2.3.4.4 hr was the primary circulating in poultry in China since 2014 and caused multiple cases of human infection [25]. A previous study showed that the A H5N1 viruses with 2.3.4.4b genes were possible introduced into China though migratory birds between September 2021 and March 2022 [24]. The viruses are still in a process of ongoing and dynamic evolution. Cui et al. [14] categorized the viruses into 16 genotypes (G1–G16) based on the analysis of 233 strains collected from wild birds and poultry from 28 countries before May 2022. Among these genotypes, four (G1, G7, G9,and G10) were found to be circulating in poultry in south of China [14]. The virus isolated from the female patient here belonged to the G10 genotype, which have been identified in Russia Far East, Japan, and Southern China since 2021.

Epidemiological investigation of the patient suggested that the patient might acquire infection from the poultry raised in her family backyard, as she had a history of direct contact with the poultry without any personal protective equipment before the onset of her illness. While the patient was identified, all poultry the patient family raised were culled and buried to prevent further spread of the virus. Due to the lack of samples, we were not able to confirm whether these poultry infected with avian influenza viruses or not. However, the presence of symptoms and deaths among the poultry suggested that they were likely infected with avian influenza viruses. Both sequence analysis and phylogenetic analysis showed the eight genes of the isolated virus had the highest homology with those of the viruses isolated from wild birds and poultry in East Asia, especially in Southern China. Different from the viruses isolated from Europe and America, the human infection with the virus JS210 maybe an event of cross-species transmission from wild birds or poultry in southern China. The A H5N1 virus bearing the clade 2.3.4.4bHA gene is still present in the wild birds and poultry in China, posing a significant threat to animal health and public health. More attention should be paid on the transmission and evolution of virus. It is necessary to continue strengthening surveillance of avian influenza virus as well as its evolution in the future.

The genetic analysis of the isolated virus from Eastern China showed that it belongs to highly pathogenic avian influenza virus. Like the clade 2.3.4.4b of A H5N1 virus that caused human infection in Europe and America [26, 27], this virus does not currently possess mutations in key sites associated with mammalian adaptation. Therefore, it is still entirely of avian origin and has not yet developed adaptive changes to infect humans. Although the clade 2.3.4.4bA H5N1 virus can occasionally spill over to infection human, it does not currently can easily transmit among people, and its threat to public health is currently low. As the clade 2.3.4.4b of A H5N1 virus continued to spread worldwide and ongoing dynamic evolution with mutations and reassortment, the risk of HPAI virus infection in human might increase. It is necessary to monitor the pandemic potential of HPAI A H5N1 in birds, mammal animal, and human.

Our study has two limitations. First, we did not collect the samples from the sick or dead poultry the patient contacted before her illness onset. Hence, we cannot confirm the exact source of infection of the patient. Second, we cannot determine if post-COVID-19 condition increase the risk of the disease severity of the patient infection with avian A H5N1 virus. The extrapolation of the results warrants more researches in the future.

In conclusion, we report a female farmer with post-COVID-19 condition and multi-comorbidity factor, who had a history of sick or dead poultry exposure, was confirmed infection with the clade 2.3.4.4b avian influenza A H5N1 virus. The pre-existing infection with SARS-CoV-2 did not increase the transmissibility of avian influenza A H5N1 but increase its morbidity and prolong the patient's hospital stay. The avian influenza A H5N1 virus bearing clade 2.3.4.4b continues to exist in poultry and has spilled over to human beings. However, there is currently no evidence of adaptive mutations in the virus that would facilitate human-to-human transmission. In the post-COVID-19 pandemic era, sporadic human cases with avian influenza A H5N1 virus are possible to occur, especially among the population who professional or occasional contact with birds or poultry. Active surveillance of individuals following a high-risk exposure to the virus without any PPE protection is strongly recommended.

## Figures and Tables

**Table 1:** Demographic characteristics and clinical laboratory test findings of a patient co-infected with A H5N1 clade2.3.4.4b virus and SARS-CoV-2.

Demographic characteristics			
Sex	Female	—	—
Age (years)	53	—	—
Occupation	Farmer	—	—
BMI	16.73 (45 kg/1.64 m)	—	—
Underlying diseases	Bronchiectasis, pulmonary nodule, and splenomegaly	—	—
History of vaccination in the past year	No influenza and COVID-19 vaccine	—	—

Clinical test findings	Date of admission to the emergency department (February 7)	Date of admission to the intensive care unit (February 10)	Normal range

White blood cell (×10^9^/L)	7.61	11.25↑	4–9 × 10^9^/L
Lymphocyte (%)	7↓	18↓	20%–40%
Neutrophil (%)	91.3↑	78.7↑	50%–70%
Hemoglobin (g/L)	114	108↓	110–150 g/L
C reaction protein (mg/L)	80.6↑	34.9↑	0–8 mg/L
Alanine transaminase (ALT) (U/L)	81.5↑	66.1↑	0–40 U/L
Aspartate transaminase (AST) (U/L)	45.9↑	102.1↑	0–40 U/L
Lactic dehydrogenase (LDH) (U/L)	292↑	381↑	109–245 U/L
B-type natriuretic peptide (pg/mL)	NA	26811.8↑	<100 pg/mL
Creatinine (*μ*mol/L)	NA	41.3↓	44–97 *μ*mol/L
Urea nitrogen (mmol/L)	NA	7.46↑	3.2–7 mmol/L
Total protein (g/L)	NA	60.9↓	60–80 g/L
Albumin (g/L)	NA	24↓	35–50 gg/L
Globulin ratio (%)	NA	0.7↓	1.5–2.5
Procalcitonin (ng/mL)	NA	1.23↑	0.1–0.5 ng/mL

Oxygenation index (PaO_2_/FiO_2_) (mmHg)	86↓	68↓	400−500 mmHg

NA: not available.

**Table 2:** Nucleotide sequence identity between A/Jiangsu/NJ210/202(H5N1) and reference strains available in GISAID.

Gene segment	Closest influenza virus relative	Nucleotide identity (%)
PB2	A/Mandarin duck/Korea/WA496/2022	99.1
	A/goose/Hunan/SE284/2022 H5N1	99.1
PB1	A/goose/Hunan/SE284/2022	99.5
	A/white-tailed eagle/Hokkaido/20220210001/2022	99.2
PA	A/duck/Bangladesh/19D1819/2021	99.1
	A/Cygnus columbianus/Hubei/128/2021	99.0
HA	A/goose/Hunan/SE284/2022 H5N1	99.7
	A/chicken/Khabarovsk/24-12V/2022(H5N1)	99.6
NP	A/goose/Hunan/SE284/2022	99.4
	A/barnacle goose/England/294620/2021	99.4
NA	A/goose/Hunan/SE284/2022 H5N1	99.2
	A/Cygnus columbianus/Hubei/121/2021	99.2
M	A/duck/Bangladesh/19D1819/2021	99.5
	A/Cygnus columbianus/Hubei/128/2021	99.4
NS	A/goose/Hunan/SE284/2022 H5N1	99.6
	A/wild duck/Hebei/SD012/2021	98.9

## Data Availability

The dataset is available upon written request to the corresponding author.

## Supplementary Materials

Supplementary 1 The phylogenetic tree of the PB1, PB2, PA, NP, MP, and NS genes coding nt sequences were generated by molecular evolutionary genetic analysis (MEGA) version 6.1 by neighbour-joining method with 1,000 bootstrap replicates. The red circle indicates human H5N1 virus isolated that we identified in 2023. The orange triangles indicate human H5N1 virus isolated in the world, 2020–2023. The black diamonds showed H5N1 viruses from environment in China, 2021−2022. Figure S1. The phylogenetic tree of the PB2 gene. Figure S2. The phylogenetic tree of the PB1 gene. Figure S3. The phylogenetic tree of the PA gene. Figure S4. The phylogenetic tree of the NP gene. Figure S5. The phylogenetic tree of the MP gene. Figure S6. The phylogenetic tree of the NS gene.

Supplementary 2 Characteristic analysis of the key amino acid mutations of A/Jiangsu/NJ210/2023 (H5N1) virus and reference viruses.
