# Supplementary material for: Severe Avian Influenza A H5N1 Clade 2.3.4.4b Virus Infection in a Human with Continuation of SARS-CoV-2 Viral RNAs
Source: Transbound Emerg Dis. 2024 May 27;2024:8819622. doi: 10.1155/2024/8819622 (PMC12019865; doi:10.1155/2024/8819622)
Supplement: Supplementary 1 — The phylogenetic tree of the PB1, PB2, PA, NP, MP, and NS genes coding nt sequences were generated by molecular evolutionary genetic analysis (MEGA) version 6.1 by neighbour-joining method with 1,000 bootstrap replicates. The red circle indicates human H5N1 virus isolated that we identified in 2023. The orange triangles indicate human H5N1 virus isolated in the world, 2020–2023. The black diamonds showed H5N1 viruses from environment in China, 2021−2022. Figure S1. The phylogenetic tree of the PB2 gene. Figure S2. The phylogenetic tree of the PB1 gene. Figure S3. The phylogenetic tree of the PA gene. Figure S4. The phylogenetic tree of the NP gene. Figure S5. The phylogenetic tree of the MP gene. Figure S6. The phylogenetic tree of the NS gene. [file 8819622.f1.pdf]

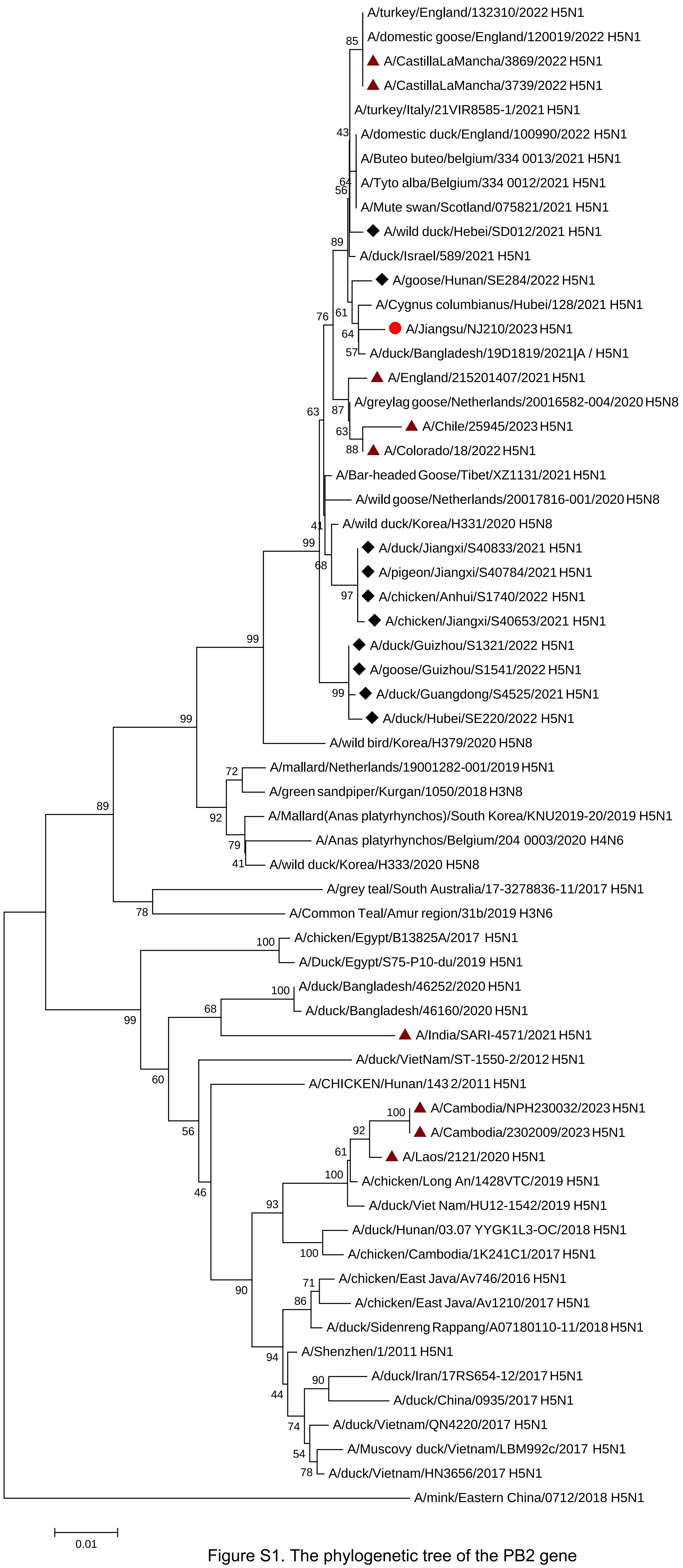

Figure S1. The phylogenetic tree of the PB2 gene

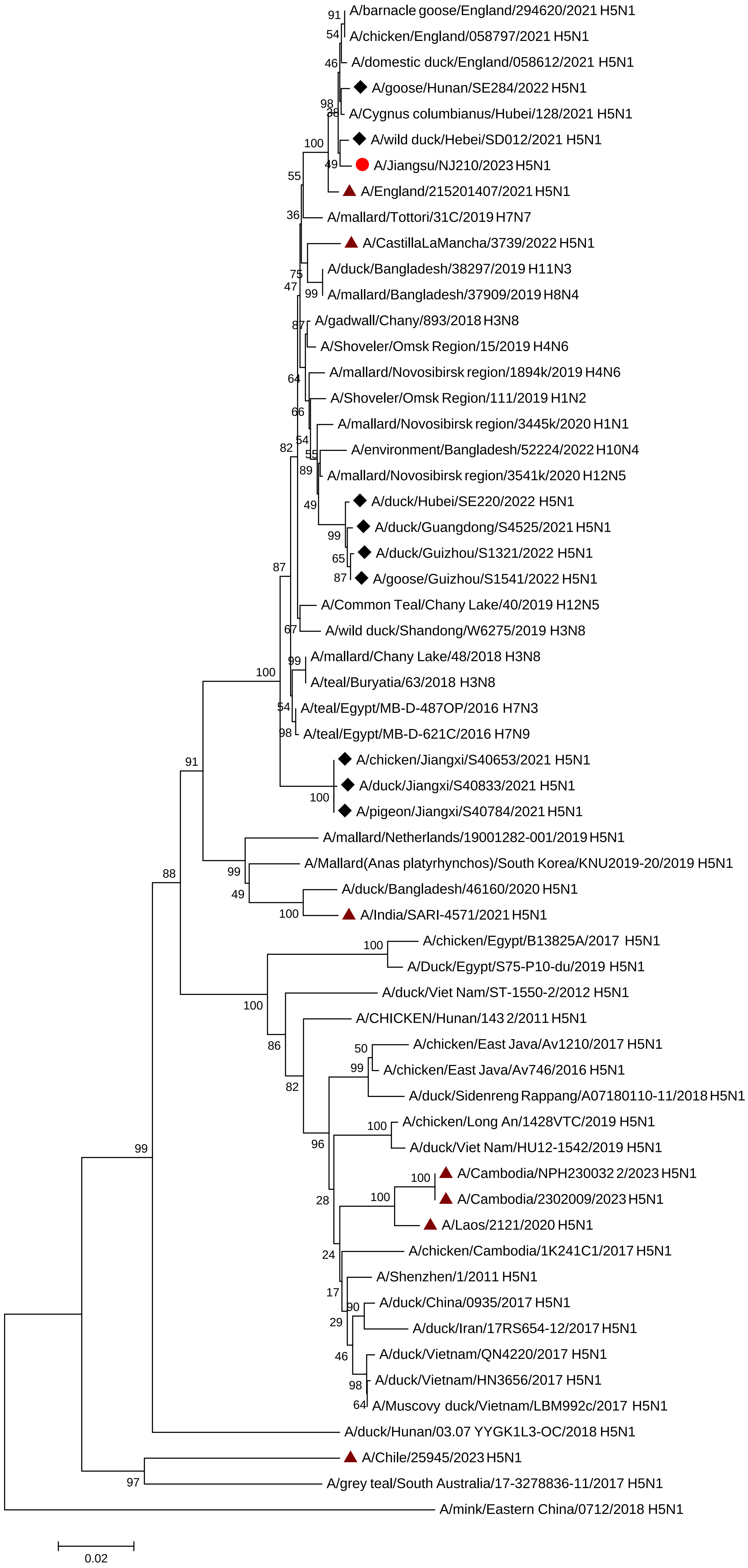

Figure S2. The phylogenetic tree of the PB1 gene

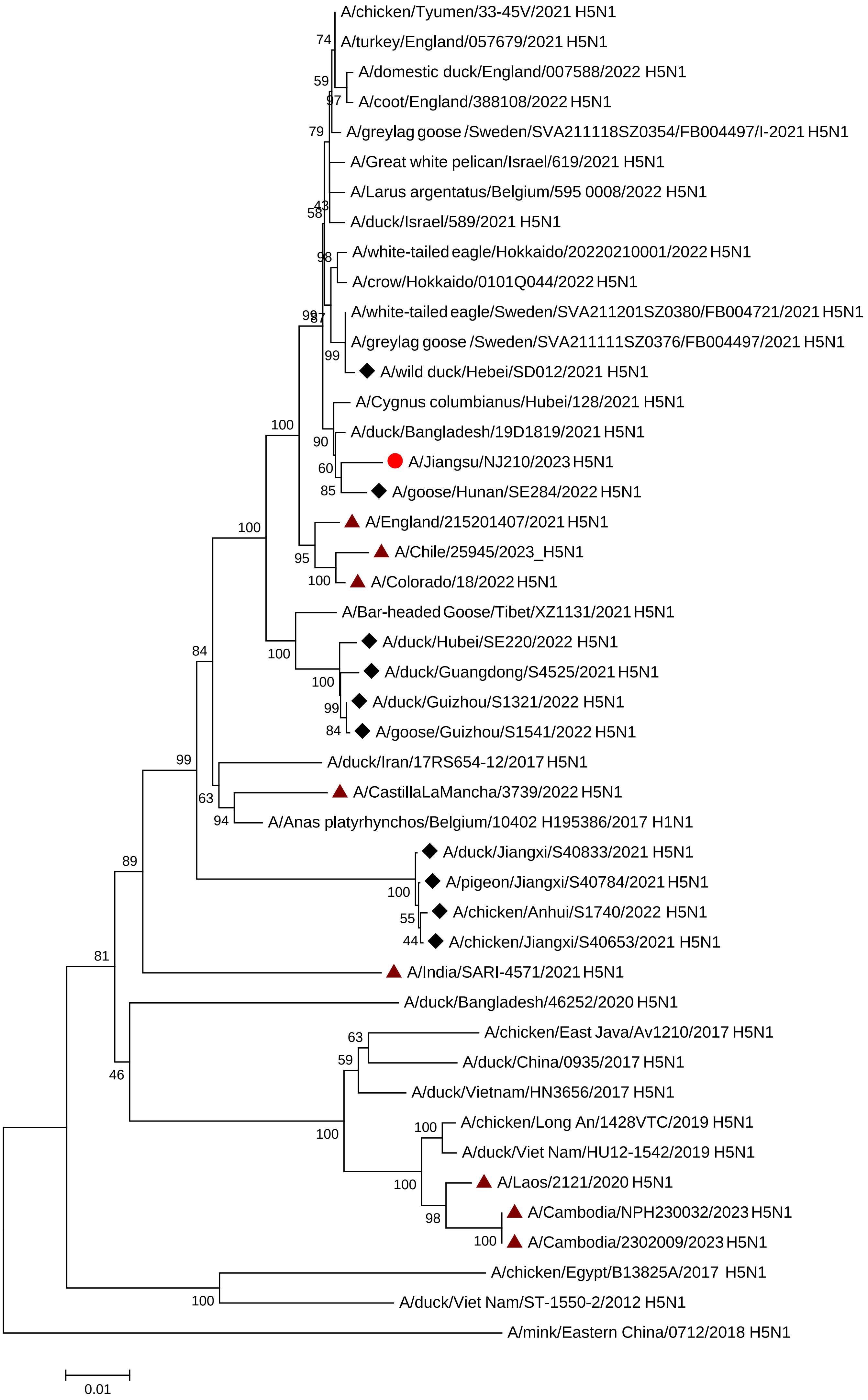

Figure S3. The phylogenetic tree of the PA gene

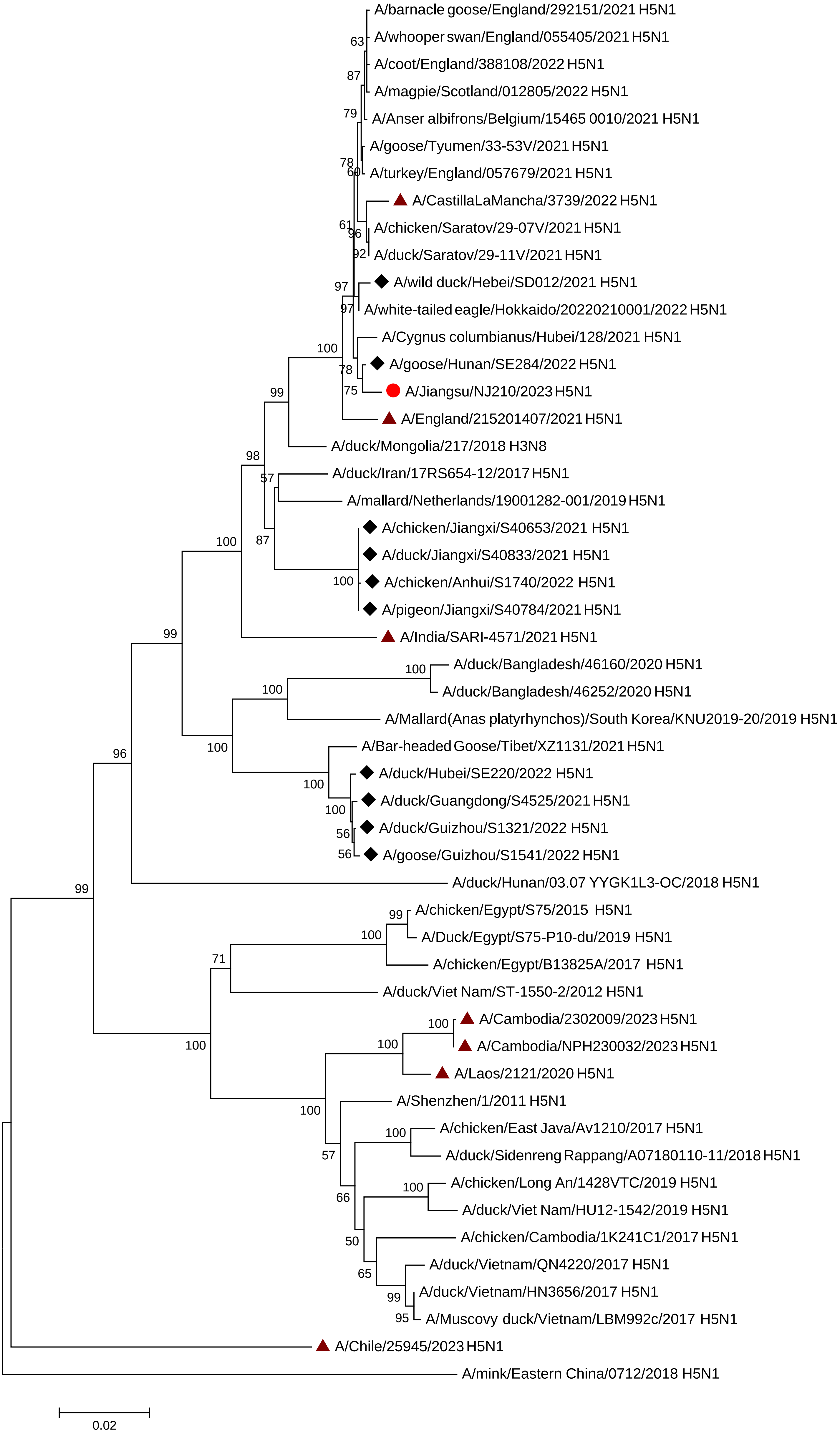

Figure S4. The phylogenetic tree of the NP gene

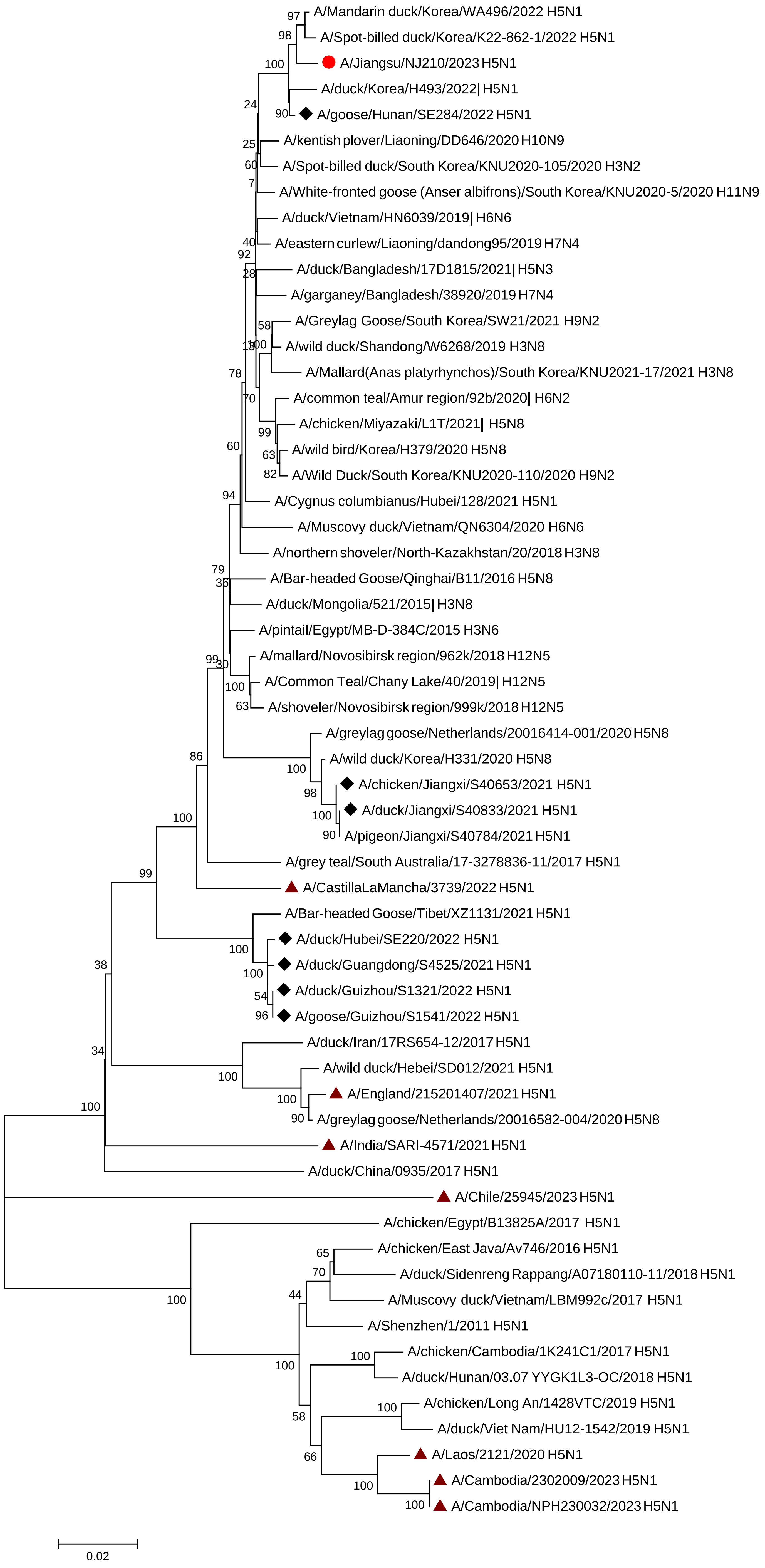

Figure S5. The phylogenetic tree of the MP gene

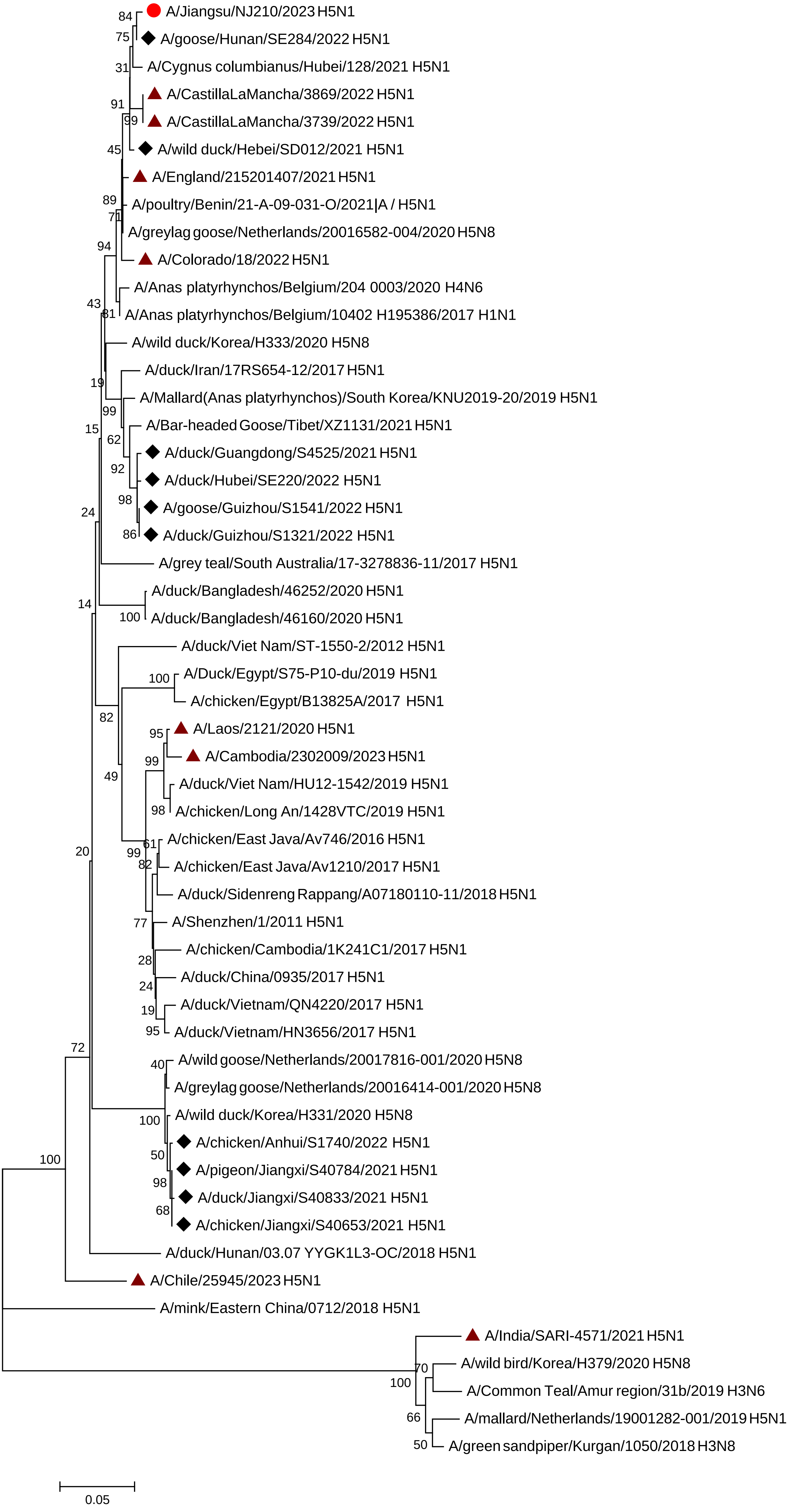

Figure S6. The phylogenetic tree of the NS gene

Figure S1-S6. The phylogenetic tree of the PB1, PB2, PA, NP, MP and NS genes coding nt sequences were generated by molecular evolutionary genetic analysis (MEGA) version 6.1 by neighbour-joining method with 1,000 bootstrap replicates. Red circle indicates human H5N1 virus isolated that we identified in 2023. The orange triangles indicate human H5N1 virus isolated in the world, 2020 – 2023. The black diamonds showed H5N1 viruses from environment in China, 2021-2022.
