# Supplementary material for: Severe Avian Influenza A H5N1 Clade 2.3.4.4b Virus Infection in a Human with Continuation of SARS-CoV-2 Viral RNAs
Source: Transbound Emerg Dis. 2024 May 27;2024:8819622. doi: 10.1155/2024/8819622 (PMC12019865; doi:10.1155/2024/8819622)
Supplement: Supplementary 2 — Characteristic analysis of the key amino acid mutations of A/Jiangsu/NJ210/2023 (H5N1) virus and reference viruses. [file 8819622.f2.docx]

**Table S1. Characteristics analysis of key amino acid mutations of A/Jiangsu/NJ210/2023 (H5N1) virus and reference viruses.**

| **Protein** | **Biological function** | **mutation** | **Strains of reference viruses^&^** | | | | | |
| --- | --- | --- | --- | --- | --- | --- | --- | --- |
|  |  |  | **js/ZJ210/23** | **gs/hn/ SE284/22** | **cas/3869/22** | **cas/3739/22** | **eng/215201407/21** | **col/18/22** |
| HA | Receptor binding sites (H3 number) | Q226L | Q | Q | Q | Q |  | Q |
|  |  | G228S | G | G | G | G | G | G |
|  |  | R229I | R | R | R | R | R | R |
|  | Cleavage site |  | REKRRKR↓G | REKRRKR↓G | REKRRKR↓G | REKRRKR↓G | REKRRKR↓G | REKRRKR↓G |
| NA | Stalk region | Deletion | No | No | No | No | No | No |
|  | Antiviral resistance | H274Y | H | H | H | H | H | H |
|  |  | N294S | N | N | N | N | N | N |
|  |  | Q136K | Q | Q | Q | Q | Q | Q |
|  |  | S246N | S | S | S | S | S | S |
|  |  | I222R/N | I | I | I | I | I | I |
| PB2 | Increased virulence in mice | T271A | T | T | T | T | T | T |
|  | Increased virulence in mice | Q591K | Q | Q | Q | Q | Q | Q |
|  | Mammalian adaptation | E627K | E | E | E | E | E | E |
|  | Increased virulence in mice | D701N | D | D | D | D | D | D |
| PB1 | Increased transmission in ferret | I368V | I | I | I | I | I | I |
| PB1-F2 | Increased virulence in mammalian | 87-90aa | 90 aa | 90 | aa | 90 | aa | 90 |
| PA | Host signature | V100A | V | V | V | V | V | V |
|  |  | S409N | S | S | S | S | S | S |
| M2 | Antiviral resistance | S31N | S | S | S | S | S | S |
| NS1 | C-terminal PED motif | 227-300aa | ESEV | ESEV | ESEV | ESEV | ESEV | ESEV |
|  | Increased virulence in mice | D92E | D | D | D | D | D | D |
|  |  | P42S | S | S | S | S | S | S |

&For the reference strains isolated from human cases with A H5N1 clade 2.3.4.4b virus and from bird.

A/Jiangsu/ZJ210/2023 (H5N1), JS/ZJ210/23,

A/goose/Hunan/SE284/2022(H5N1), GS/hn/ SE284/22,

A/CastillaLaMancha/3869/2022,cas/3869/22,

A/CastillaLaMancha/3739/2022,cas/3739/22,

A/England/215201407/2021,eng/215201407/21,

A/Colorado/18/2022,col/18/22
